# An Unusual Presentation of Right-Sided Sciatica with Foot Drop

**DOI:** 10.1155/2016/9024368

**Published:** 2016-04-27

**Authors:** Fergus J. McCabe, John P. McCabe

**Affiliations:** ^1^School of Medicine, NUI Galway, Galway, Ireland; ^2^Department of Trauma & Orthopaedics, Galway University Hospitals, Galway, Ireland

## Abstract

Rarely, sciatica is of extraspinal aetiology. By compressing the sciatic nerve, swelling of the short external rotators of the hip can cause sciatica. Uncommon anatomical relationships between the sciatic nerve and local muscles may potentiate this compressive effect. In this report, we describe the presentation of right sciatica and foot drop resulting from both extreme local constriction and unusual anatomical variation of the right sciatic nerve.

## 1. Introduction

The sciatic nerve is the major motor and sensory nerve of the lower limb [1]. In the vast majority of cases, the sciatic nerve enters the lower limb via the greater sciatic foramen, passing below piriformis [2]. The nerve, or its constituent parts, can have uncommon anatomical relationships with the piriformis muscle [2]. Sciatica is defined by the Oxford Medical Dictionary as “pain radiating from the buttock into the thigh, calf and occasionally the foot,” occurring in the territory of the sciatic nerve [1]. Spinal disc herniation is the most common cause of sciatica, with extraspinal infrequent causes [3]. Piriformis syndrome is an extraspinal entrapment neuropathy in which the piriformis muscle compresses the sciatic nerve, causing sciatica [4]. Other gluteal muscles, including obturator internus, may constrict the sciatic nerve, producing similar effects [5].

In this case study, we present an unusual case of sciatica and foot drop, of multifactorial aetiology.

## 2. Case Report

A 62-year-old man presented with pain and numbness in the right gluteal area which radiated down the posterior aspect of the right thigh and leg.

This began gradually and then spread down the back of the right thigh, eventually starting to limit his ability to stand, walk, sit, and in particular drive. There was no specific history of trauma to the area. Sitting aggravated the pain. The pain and discomfort were sharp, continual, and severe, disturbing the man's sleep pattern and hindering his quality of life. He also complained of constipation which began in the months after a prostatectomy. The man denied having any undue back pain and was otherwise in good health. An MRI taken 6 days previously showed intervertebral disc degeneration but no evidence of disc herniation. He had been prescribed combined 500 mg paracetamol and 30 mg codeine for pain relief. The man was a self-employed painter and was overweight, with a BMI of 28. He rarely drank and never smoked.

Examination revealed an antalgic gait affecting the right leg, indicating pain with weight bearing on the right side. Right hip range of motion was normal. Significantly, he was remarkably tender immediately lateral to the right ischial tuberosity, suggesting that the cause was located in the gluteal area itself. He had a grossly restricted straight leg raising test, at 25°.

The man underwent an MRI scan focusing on his hips, with particular emphasis on the path of the right sciatic nerve in the right gluteal area and around the piriformis muscle. This demonstrated inflammation of the piriformis, obturator internus, and gemelli muscles on the right side and of the right sciatic nerve. There was no evidence of abscess or fracture in the area. Blood analysis showed no evidence of sepsis and his C-reactive protein levels were normal. The patient was discharged four days later with prescribed gabapentin (300 mg twice daily) and celecoxib (100 mg twice daily), with a view for future surgical decompression of the nerve if the pain persisted.

**Figure 1:**
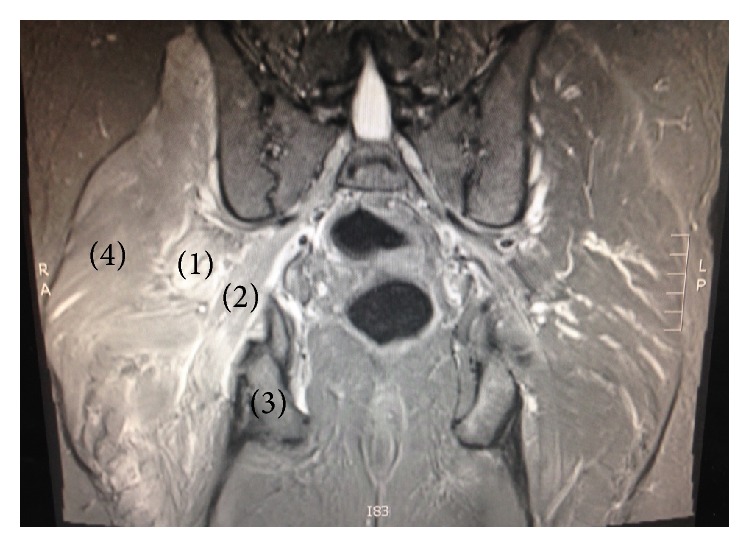
Signal abnormality of the right short external rotators on coronal section of T2-weighted MRI. (1) Piriformis. (2) Obturator internus. (3) Ischium. (4) Gluteus maximus.

**Figure 2:**
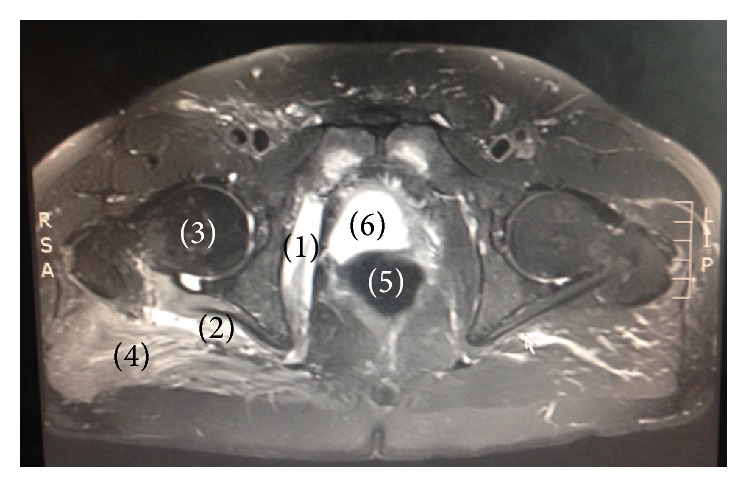
Signal abnormality of the right short external rotators on axial section of T2-weighted MRI. (1) Obturator internus. (2) Quadratus femoris. (3) Head of femur. (4) Gluteus maximus. (5) Rectum. (6) Bladder.

The man returned 6 weeks later with nonimprovement of his symptoms, despite some relief with medication. A subsequent MRI displayed unchanged swelling of the right piriformis, obturator internus, and sciatic nerve (Figures 1 and 2) but new tumefaction of gluteus medius and minimus on the same side. At this point the patient revealed that, following a prostatectomy, he developed constipation and began sitting on his right buttock while on the toilet in order to pass a normal bowel movement. Based on deteriorating clinical status and new inflammation of gluteus medius and minimus, it was decided to carry out exploration of the right sciatic nerve and biopsy of the right short external rotators. Prior to surgery, a right, complete foot drop developed (grade 0/5 dorsiflexion).

**Figure 3:**
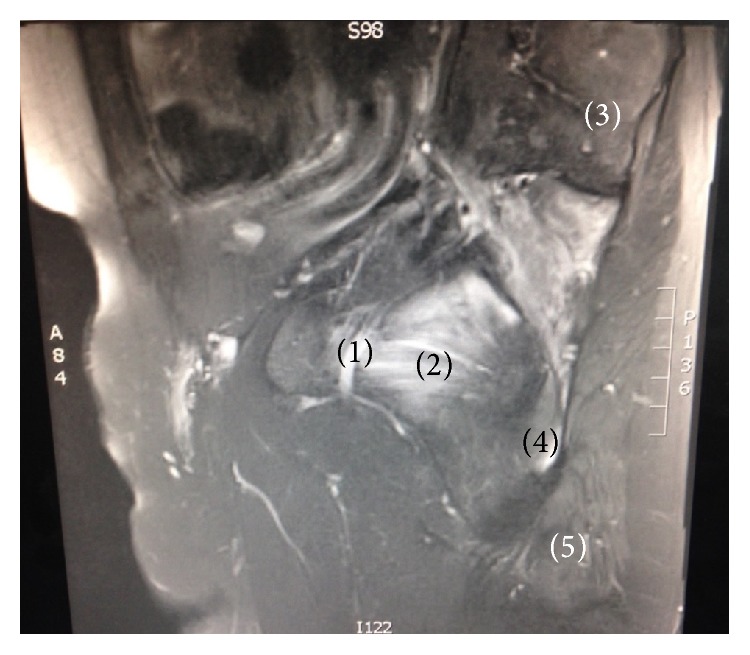
Oblique parasagittal section of T2-weighted MRI displaying passage of the right sciatic nerve above the piriformis muscle. (1) Sciatic nerve. (2) Piriformis. (3) Sacroiliac joint. (4) Ischium. (5) Ischial tuberosity.

**Figure 4:**
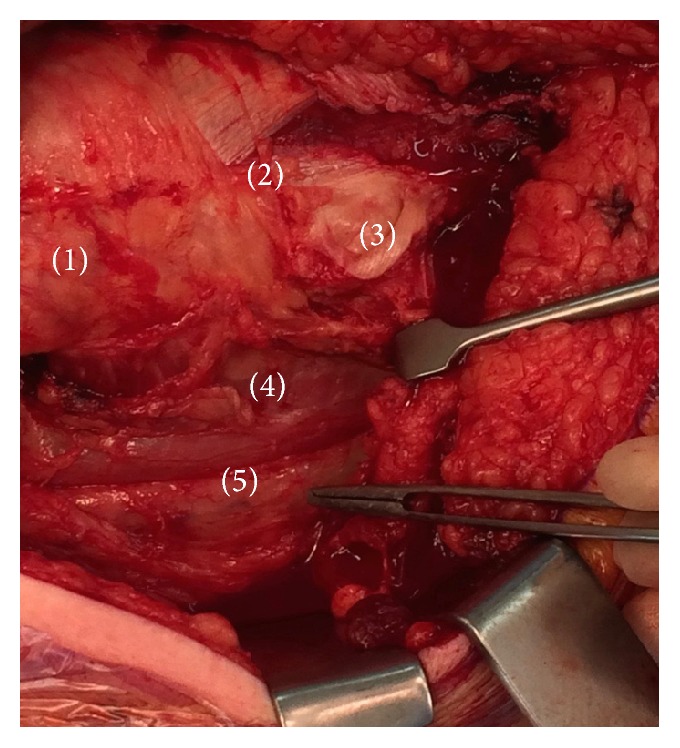
Intraoperative image before resection of the short external rotators of the right hip. (1) Greater trochanter. (2) Vastus lateralis. (3) Insertion of the tendon of gluteus maximus. (4) Short external rotators of the right hip (deep to sciatic nerve). (5) Swollen sciatic nerve.

**Figure 5:**
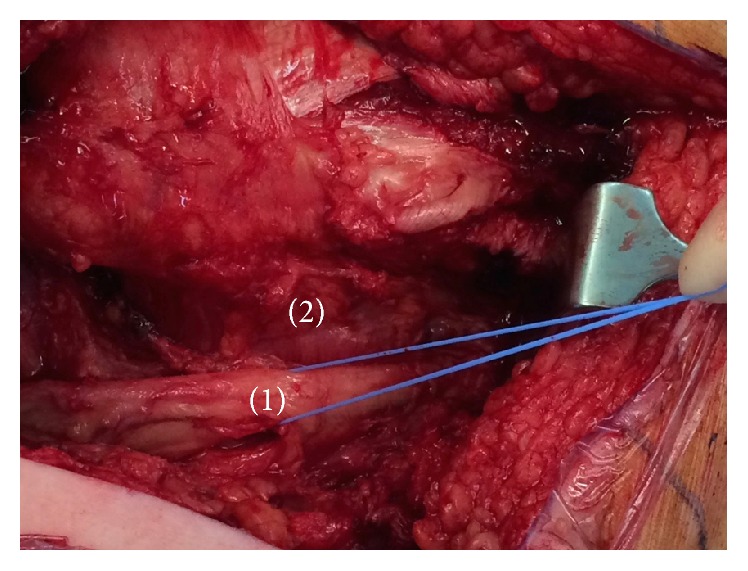
Intraoperative image after resection of the short external rotators of the right hip. (1) Decompressed sciatic nerve. (2) Area previously occupied by short external rotators of the right hip.

The right hip exploration exposed gross swelling and dullness of colour of the piriformis muscle and the gemelli-obturator internus complex. The sciatic nerve was explored back to the greater sciatic foramen to show both peroneal and tibial roots of the sciatic nerve exiting together above the piriformis muscle (Figure 3), one of the rarest anatomical variations [2]. The nerve itself was extremely tight, with an exceptionally broad diameter (Figure 4). The nerve was decompressed (Figure 5) and the piriformis and conjoint tendon were biopsied for histopathological analysis. Upon analysis, both samples showed identical type II muscle fibre atrophy, suggestive of chronic muscle denervation without reinnervation. There was no evidence of myositis or dystrophy. The man was discharged 3 days later.

In the weeks following surgery, the man's pain significantly improved, requiring substantially less analgaesia. Additionally, the numbness subsided and sensation returned to the right foot. However, the foot drop did not resolve, reflecting permanent, motor sciatic nerve damage. The man received a foot drop splint and physiotherapy.

## 3. Discussion

Sciatica is most frequently a symptom indicative of spinal disc herniation and seldom has an extraspinal aetiology [3]. In this instance, the source was extraspinal. His sciatica and foot drop were caused by stricture of the right sciatic nerve in the right gluteal area, arising from extreme swelling of the short external rotators of the hip. Undoubtedly, this is quite rare.

Constriction of the sciatic nerve in the gluteal area, by piriformis (piriformis syndrome), can produce sciatica [4]. Piriformis syndrome accounts for a mere 6–8% of sciatica cases in the USA [4]. Meknas et al. showed that certain suspected cases of piriformis syndrome may be due to sciatic nerve crushing by obturator internus muscle, without piriformis involvement [5]. This case is particularly compelling as the sciatica resulted not only from compression by piriformis, but also by obturator internus, the gemelli, and to a lesser extent gluteus medius and minimus. Therefore, this example cannot be classified as a simple incidence of piriformis syndrome, but rather a form of deep gluteal syndrome [6]. While compression from both piriformis and obturator internus can cause pain [4, 5], the presence of sciatic nerve irritation by multiple muscles of the gluteal compartment, without any history of acute trauma, leads us to believe that this individual circumstance is unique.

The pathophysiological basis of the muscle inflammation is unclear. We believe the origin of the muscle swelling to be the patient's tendency to preferentially sit on his right buttock to ease defaecation, subsequent to a prostatectomy. There were no history of trauma to the area, no evidence of myositis, dystrophy, or normal inflammatory markers, and no other identifiable causes of muscle irritation. The short external rotators became sequentially involved, with initial inflammation of piriformis and the obturator-gemelli complex and progression to gluteus medius and minimus, 6 weeks later. This finding, coupled with the increased pain and foot drop development, indicated a progressive pathology, requiring surgical intervention [3].

Another pivotal aspect was the increased tension of the sciatic nerve, due to its irregular anatomical course. This man's sciatic nerve had one of the least common anatomical relations with the piriformis muscle. A study of 294 cadaveric lower limbs in 2014 showed only 0.3% prevalence (1 limb) of passage of both peroneal and tibial parts of the sciatic nerve above piriformis, as was the case here [2]. Furthermore, Smoll's meta-analysis of the topic found that only 5 of 6,062 cadaveric limbs (0.082%) exhibited this anatomical anomaly [7]. Undeniably, the remarkable, protracted route of the sciatic nerve augmented its intrinsic tension, thus exacerbating the entrapment neuropathy.

A combination of both local compression by muscle and rare anatomical variation led to this man's symptoms. This case offered a valuable exercise in critical thinking and prudence. While initial findings pointed to an overt, spinal explanation, careful attention to minute detail led to the correct diagnosis, irrespective of its atypicality.
